# Editorial: Use of radiation therapy for hematological malignancies

**DOI:** 10.3389/fonc.2026.1781001

**Published:** 2026-02-20

**Authors:** Carla Hajj, Dana L. Casey, Brandon S. Imber

**Affiliations:** 1 Department of Radiation Oncology, Cleveland Clinic Abu Dhabi, Abu Dhabi, United Arab Emirates; 2 Department of Radiation Oncology, University of North Carolina School of Medicine, Chapel Hill, NC, United States; 3 Department of Radiation Oncology, Memorial Sloan Kettering Cancer Center, New York, NY, United States

**Keywords:** combined modality therapy, dose and fractionation, hematologic malignancies, radiation therapy, radiogenomics, lymphoma

This Research Topic assembles nine contributions that collectively highlight the remarkable breadth and rapid evolution of radiation therapy in the management of hematologic malignancies. Although these cancers are historically considered highly radiosensitive, the modern role of radiation therapy is being reshaped by advances in systemic therapy, imaging, immunotherapy, and cellular therapies. Across the included articles, several unifying and forward-looking themes emerge: refinement of dose and fractionation, innovative delivery techniques to improve toxicity profiles, optimal integration of radiation therapy with novel therapeutics, and deeper biologic insights to increasingly enable personalization. These studies underscore a renewed appreciation for radiation therapy not as a static modality, but as a dynamic and adaptive component of modern hematologic cancer care.

In indolent B cell lymphomas, the combination of low dose radiation and anti-CD20 therapy continues to generate significant interest. Lake et al. present an institutional experience incorporating single agent rituximab with 4 Gy ultra-low dose radiotherapy, delivered adaptively with repeat courses as needed. Their cohort demonstrated excellent early responses, with a 90% overall response rate and 2-year in field control exceeding 90%. These outcomes reinforce ultra-low dose radiotherapy as an effective, well tolerated, and importantly, repeatable modality. Durable disease control and high overall survival were achieved without histologic transformation, further supporting combined biologic and radiation-based approaches in the management of indolent lymphoma.

Primary cutaneous lymphomas represent another domain where radiation therapy plays a central role. Specht provides an expert overview of total skin electron beam therapy (TSEBT), emphasizing the unique physical properties of electrons that allow safe treatment of superficial, widespread disease. The shift from historical higher total doses to contemporary low dose regimens, which can be also delivered repeatedly, has expanded the therapeutic window and improved long-term palliation. Attempts to replicate total skin irradiation with photon-based techniques such as tomotherapy have resulted in excessive dose to marrow and deeper structures, highlighting the continued importance of electron-based approaches. Ongoing research into dose, technique optimization, and integration with systemic therapies is likely to further enhance outcomes. This is particularly true given relatively short durability of response after low dose TSEBT, highlighting a need for better maintenance therapies.

Urgent neurologic presentations remain among the most challenging scenarios in the care of patients with lymphoma, leukemia, and myeloma. Tringale et al. provide a comprehensive and practical guide to urgent and emergent central nervous system directed radiation therapy, including indications, simulation strategies, dose ranges, and sequencing with systemic and intrathecal therapies. Their review highlights radiation therapy as the most rapid and reliable modality for salvaging cranial neuropathies and neurologic deficits. Importantly, they emphasize the need for early multidisciplinary evaluation, since certain combinations of radiation therapy with systemic or intrathecal agents can amplify neurotoxicity. With the increasing use of chimeric antigen receptor T cell therapy and other novel agents, the role of focal radiation therapy or craniospinal irradiation as bridging or consolidative strategies requires further prospective investigation.

Biologic and genomic factors that influence radiosensitivity are explored in depth by Wijetunga et al. who review the genomic and epigenomic landscape of hematologic malignancies as it relates to radiation response. By tracing key discoveries involving genes such as TP53 and ATM, the authors outline how genomic instability and defects in DNA repair intersect with radiation sensitivity. While the field of radiogenomics remains in its early stages, there is clear potential for genomic markers to guide radiation dose personalization, predict toxicity, and refine integration with systemic therapies. The development of standardized protocols, clinical decision-support frameworks, and prospective validation studies will be essential to translating these insights into routine practice.

Three contributions focus on rare extranodal lymphomas. In their institutional experience and literature synthesis of primary hepatic lymphoma, Ma et al. describe a heterogeneous disease that is most often treated with systemic therapy but where radiation therapy appears particularly effective for indolent histologies. Their findings suggest that low dose radiation therapy can provide excellent disease control with minimal toxicity in carefully selected patients. Similarly, El Khoury et al. review the sparse literature on primary esophageal lymphoma, a rare diagnosis without a clear consensus on management. Across published reports, multimodality treatment that incorporates chemotherapy, radiation therapy, and occasionally surgery emerges as the most effective approach, tailored to histologic subtype and clinical presentation. Manzar et al. examine outcomes in diffuse large B cell lymphoma involving the gastrointestinal tract, a relatively rare presentation that has not been well characterized in contemporary real-world cohorts. In more than two hundred patients, combined modality therapy that incorporates radiation therapy achieved excellent long-term survival with minimal late toxicity. Notably, stomach-directed radiation therapy was associated with improved survival, and consolidative radiation therapy appeared beneficial in bulky disease with incomplete initial responses. These findings support the value of radiation therapy as part of risk adapted combined modality treatment, including in the era after the introduction of rituximab. These studies reinforce the important role of radiation therapy in rare extranodal sites where prospective data are limited.

Advances in radiation delivery technology are highlighted in the analysis by Hui et al., who compare volumetric modulated arc therapy total body irradiation with traditional two-dimensional total body irradiation in a cohort of two hundred patients. Volumetric modulated arc therapy total body irradiation achieved substantial sparing of organs at risk and translated into significantly reduced rates of pneumonitis, nephrotoxicity, nausea, skin toxicity, and graft versus host disease, without compromising overall or progression free survival. These findings strengthen the rationale for modernizing total body irradiation techniques, particularly in pediatric and young adult populations, in whom reduction of long-term toxicity is paramount.

Finally, Danish et al. evaluate patients with relapsed or refractory non-Hodgkin lymphoma receiving CD19 chimeric antigen receptor T cell therapy in order to determine how many would be candidates for bridging radiation therapy based on disease distribution. Nearly half of patients met high risk criteria for early relapse while also having a limited number of lesions that were suitable for a standard radiation plan. As cellular therapies expand, identifying patients who may benefit from bridging radiation therapy, particularly those with bulky, extranodal, or very metabolically active disease, will be critical for optimizing outcomes and reducing early treatment failure.

Taken together, these nine contributions illustrate the expanding and increasingly sophisticated role of radiation therapy across the spectrum of hematologic malignancies. Advances in technique, biology, integration with systemic therapy, and personalization are shaping an exciting and rapidly evolving landscape. We hope this Research Topic serves as a valuable resource for clinicians and researchers dedicated to improving outcomes for patients with hematologic cancers.

